# Interplay between HSF1 and p53 signaling pathways in cancer initiation and progression: non-oncogene and oncogene addiction

**DOI:** 10.1007/s13402-019-00452-0

**Published:** 2019-06-10

**Authors:** Agnieszka Toma-Jonik, Natalia Vydra, Patryk Janus, Wiesława Widłak

**Affiliations:** Maria Skłodowska-Curie Institute – Oncology Center, Gliwice Branch, Wybrzeże Armii Krajowej 15, 44-101 Gliwice, Poland

**Keywords:** HSF1, p53, Transcription factors, Oncogenesis

## Abstract

**Background:**

The p53 and HSF1 transcription factors are key players in cellular responses to stress. They activate important signaling pathways triggering adaptive mechanisms that maintain cellular homeostasis. HSF1 is mainly activated by proteotoxic stress, and its induction leads to the synthesis of chaperones that provide proteome integrity. The p53 protein, which is primarily activated in response to DNA damage, causes cell cycle arrest allowing for DNA repair or directs cells to apoptosis, thereby maintaining genome integrity. Both signaling pathways are also involved in neoplastic transformation and tumor progression. Loss of tumor suppressor abilities of the wild-type p53 protein results in oncogenesis, whereas proper HSF1 action, though non-oncogenic itself, actively supports this process.

**Conclusions:**

Here, we describe in detail the interplay between the p53 and HSF1 signaling pathways, with particular emphasis on the molecular mechanisms involved, as well as their importance for normal cellular behavior, cancer development, the effectiveness of anti-cancer therapies and their toxicity. Detailed knowledge of the complex interplay between HSF1 and p53 may form a basis for the design of new protocols for cancer treatment.

## Introduction

Living cells possess different mechanisms that allow them to adapt to changes in their environment. These cellular mechanisms involve alterations in metabolism, as well as in signal transduction, transcription, translation, protein packaging and/or release. The initial cellular response is aimed at defense against a harmful factor. If the damaging signals are too severe and cause irreversible injury, cells activate death signaling pathways. The activation of protective or destructive pathways depends to a large extent on the nature and duration of the stress, as well as the cell type involved. The final fate of the stressed cell depends on the interplay between these responses.

One of the main pro-survival responses is activation of the Heat Shock Response (HSR). Originally, it was described as a biochemical reaction of the cell in response to elevated temperatures [1]. Later it was found that this response may also be activated through oxidative stress, heavy metal exposure, etc. The HSR is thought to be induced by damaged (misfolded and/or aggregated) proteins, although changes in membrane surface may also act as initiating events [2]. To counteract these events, cells may increase the expression of chaperone proteins, especially Heat Shock Proteins (HSPs), which participate in the refolding of misfolded (denatured) proteins. HSPs have also been found to be involved in the acquisition of thermotolerance, a state in which cells become more resistant to toxic insults [3]. During initiation of the HSR, global gene transcription and protein synthesis are halted, presumably to diminish the level of misfolded proteins in the cell, whereas the expression of stress-responsive genes is increased due to selective activation of some transcription factors, i.e., Heat Shock transcription Factors (HSFs). In mammals, the HSF family includes several members (HSF1, HSF2, HSF3, HSF4, and loosely related HSF5, HSFX and HSFY), which differ in their pattern of expression and differently affect the expression of stress-responsive genes [4, 5].

## HSF1 plays an important role in cytoprotection

The transcription factor HSF1 (Heat Shock Factor 1) plays a predominant role in the cellular response to heat shock. It is mainly activated by elevated temperature, but may also be activated by other factors causing proteome imbalance, i.e., proteotoxic stress including oxidative stress, heavy metals, salicylates and acidification of the environment. HSF1 is a multidomain protein (for details, see [6]). In normal cells under physiological conditions, it is predominantly situated in the cytoplasm in the form of an inactive monomer. During stress, HSF1 acquires trimerization capabilities, undergoes phosphorylation and translocates to the nucleus. After binding to target sequences, i.e., HSEs (Heat Shock Elements), it induces the expression of HSF1-dependent genes. Although HSF1 phosphorylation may occur on many serine residues, the phosphorylation of serine 326 (S326) seems to be crucial for achieving full protein activation [7].

**Fig. 1:**
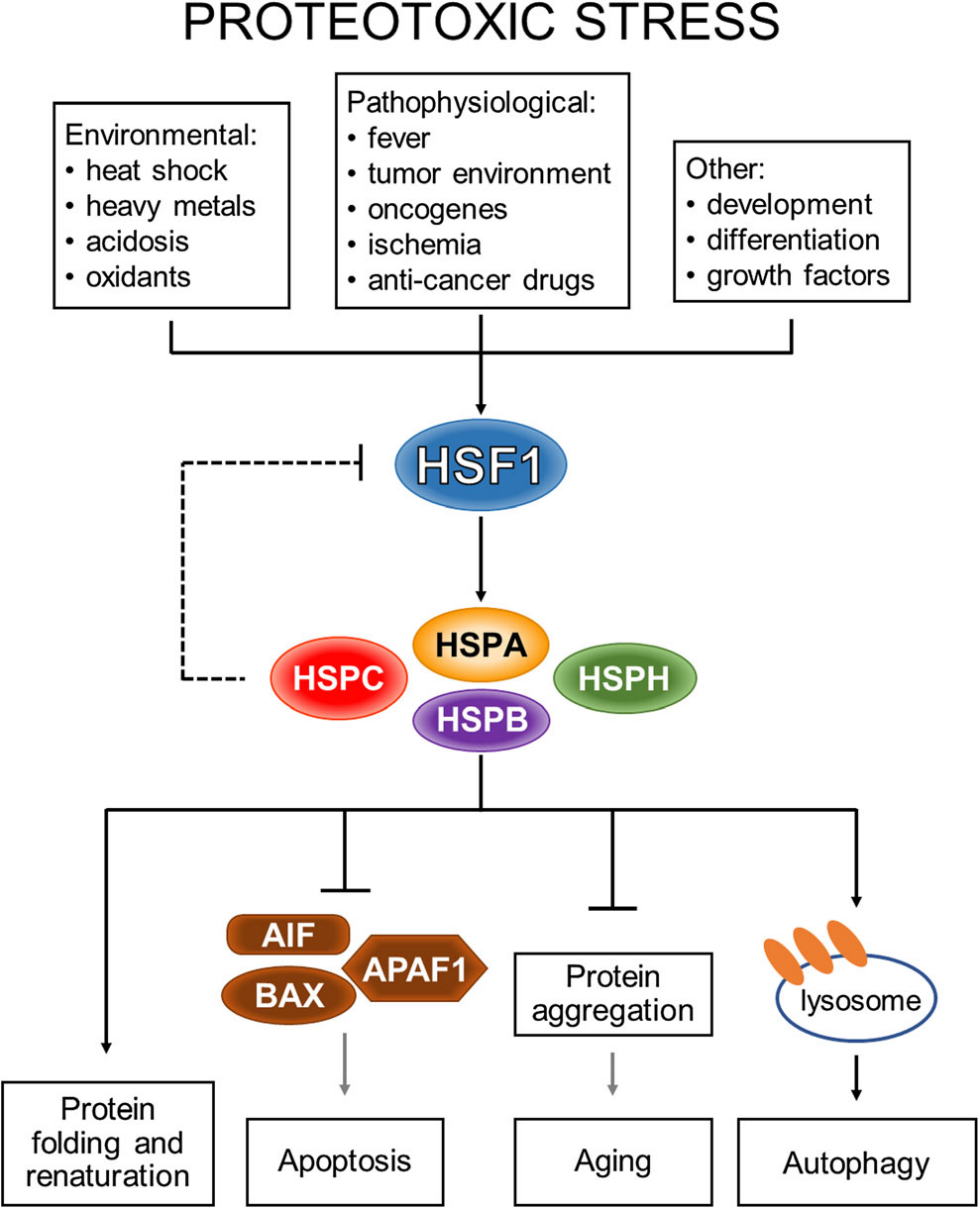
**The heat shock response (HSR)**. The transcription factor HSF1 can be activated by many proteotoxic stressors, which leads to Heat Shock Protein (HSP) expression. Newly synthesized HSPs act as cytoprotective factors that enable the refolding of damaged proteins, inhibit apoptosis by interaction with apoptotic regulators (such as BAX, AIF and APAF1) and prevent protein aggregation, thus protecting cells from senescence and aging. HSPs also stabilizes the lysosomal membrane, stimulating the removal of irreversibly impaired proteins in the autophagy process

The main targets of activated HSF1 are genes encoding highly evolutionary conserved chaperones, primarily belonging to HSP families, i.e., HSPH (HSP110), HSPC (HSP90), HSPA (HSP70), DNAJ (HSP40) and HSPB (small HSPs) [8, 9]. The cytoprotective action of HSPs is based on binding to misfolded proteins and restoring their correct conformation. HSPs prevent excessive protein denaturation and aggregation as well as repress apoptosis and senescence while stimulating autophagy [10, 11] (Fig. 1). Therefore, HSF1 can be considered as “the guardian of the cellular proteome”. HSF1-deficient cells exhibit increased heat sensitivity and lack the ability to develop thermotolerance due to diminished HSP synthesis [12, 13]. Moreover, multiple experimental models have revealed a role of HSF1 in other biological processes far beyond the regulation of HSP expression. Lack of HSF1 in mice has, for example, been found to result in an abnormal sperm morphology and its reduced production, in defects in oogenesis and preimplantation development, in defects in the placenta and increased prenatal lethality as well as in postnatal growth retardation. In adults, redox homeostasis and antioxidative defenses, immune response, motor activity, smell, hearing, memory, and other senses have also been found to be affected [14, 15]. HSF1 activity is essential for numerous signaling pathways including p53 signaling.

## p53: Guardian of the genome

The human p53 protein is encoded by the *TP53* gene belonging to a highly conserved gene family, which also includes *TP63* and *TP73* [16]. All family members can generate multiple transcript variants and protein isoforms that vary in their functional properties. The full-length p53 protein isoform consists of 393 amino acids and encompasses several structural-functional domains: a N-terminal transactivation domain, a central domain responsible for binding to DNA and a C-terminal tetramerization domain [16].

**Fig. 2:**
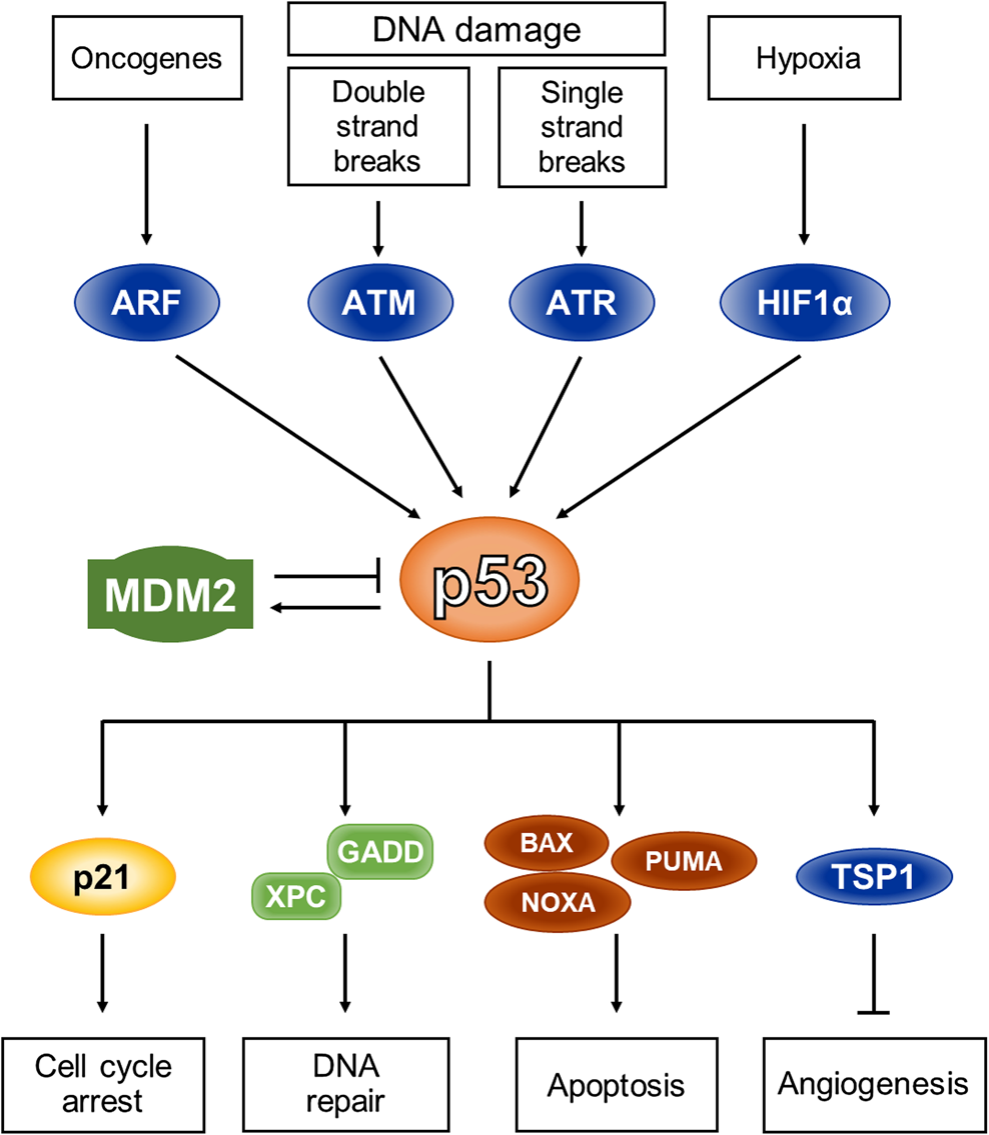
**p53 signaling**. p53 can be activated by genotoxic stress as well as by signaling induced by activated oncogenes and stress factors such as hypoxia and nucleotide depletion. Its transcriptional activity is repressed by MDM2 (encoded by the p53-responsive gene). Activation of p53 typically leads to p21-mediated cell cycle arrest and DNA repair (which may be mediated by XPC and GADD) to allow cell survival. When appropriate repair is not possible, p53 stimulates the expression of pro-apoptotic proteins to discard the damaged cell. Another role of p53 is the regulation of thrombospondin (TSP1) expression, which inhibits angiogenesis

As a transcription factor, p53 plays a key role in the cellular response to stress. It can be induced by stress factors, such as DNA damage, hypoxia, ribonucleotide depletion and oncogene activation, that deregulate signal transduction pathways and cellular proliferation (Fig. 2). The p53 protein is labile and its abundance and activity are strictly controlled by E3 ubiquitin ligases, primarily MDM2. Its regulation is based on negative feedback since transcription of the *MDM2* gene can be activated by p53. Under normal conditions, the p53 protein level is regulated by a balance between its synthesis and degradation. Inactive (non-phosphorylated) p53 protein associates with MDM2 which, in turn, stimulates efficient p53 folding, ubiquitination and degradation by the proteasome [17]. In addition, MDM2 controls nucleocytoplasmic shuttling of p53. In response to cellular stress, the stability of the p53 protein is markedly increased, leading to a quick accumulation in the nucleus. The p53 protein can be phosphorylated by specific kinases, including those from the MAPK (mitogen-activated protein kinase) family members, CHEK1 and CHEK2 (checkpoint kinases), ATM and ATR (serine/threonine kinases) and others, thereby inducing conformational changes and a release of MDM2. These changes enable p53 to form a tetramer and to initiate its action as a transcription factor [18]. The p53 protein can directly activate target genes that mediate cell cycle arrest, DNA repair, apoptosis, angiogenesis, metabolism, autophagy, translation control and feedback mechanisms (Fig. 2). The downregulation of genes by p53 is indirect and is primarily mediated by p21 (CDKN1A, the cyclin-dependent kinase inhibitor), as well as by the retinoblastoma protein (RB1) [19].

Wild-type p53 is often called “the guardian of the genome” because it prevents the transmission of genetic damage to daughter cells, thereby protecting multicellular organisms from disrupted and uncoordinated cellular proliferation. Once activated, it can promote cell cycle arrest, DNA repair, autophagy and senescence, or the elimination of cells via apoptosis (when the DNA damage is unrecoverable). Consequently, lack of p53 expression or its adverse action may lead to neoplastic transformation and tumor progression. Thus, wild-type p53 is classified as a tumor suppressor, although its mutant forms are considered oncogenic [16, 20, 21].

## Interplay of HSF1 and p53 in the cellular response to stress

Both wild-type p53 and HSF1 play key roles in genotoxic and proteotoxic stress responses, respectively, that enable cells to survive or induce the elimination of damaged cells. HSF1 and p53 can directly interact with each other in the regulation of transcription. In addition, signaling pathways induced by both transcription factors may interact at many levels. As HSF1 is required for p53-mediated transcription, cell-cycle arrest, DNA repair or apoptosis in response to p53 activating agents [22–24], wild-type p53 is required for HSF1 activation and HSP accumulation after heat shock [25]. HSF1 can form a steady-state complex with the DNA damage kinases ATR and CHEK1 to affect p53 phosphorylation [22], but other mechanisms of supportive HSF1 action may also exist. On the other hand, it has been found that cell cycle arrest induced by heat shock is dependent not only on HSF1 [26, 27], but also on proper function of the p53 protein (that becomes accumulated in the nucleus and increases the p21 level after heat shock) [28–30].

HSF1 influences p53 protein turnover via HSF1-dependent chaperones. They can directly interact with p53 or can modulate proteasome activity, which is crucial for the degradation of p53. As a result, the wild-type p53 half-life has been found to be slightly longer in immortalized HSF1−/− mouse embryonic fibroblasts (MEFs) than in HSF1+/+ cells [31–33]. Interestingly, wild-type and mutant p53 differentially interact with HSPs, which has consequences for their stability. Wild-type p53 interacts with some HSPs only transiently, while structural p53 mutants (e.g. R175H) can create a more stable multi-chaperone complex with DNAJB1 (HSP40), HSPA1 (HSP70) or HSPA8 (HSC70), STIP1 (HOP) and HSP90AA1 (HSP90) [34–38]. Such a complex has been found to be crucial for stabilizing mutant p53 and increasing its half-life in cancer cells [35, 39, 40]. It has also been postulated that HSP90 stabilizes p53 through its interaction with MDM2 and inhibition of its ubiquitin ligase activity, thus preventing p53 degradation [41, 42].

Another level of interaction between HSF1 and p53 relates to transport of p53 to the nucleus. Activated p53 protein is transported into the nucleus along the microtubule cytoskeleton through interactions with the dynein motor protein [43] and functional HSF1 seems to be required for this process. Neither the interaction of microtubules with activated p53, nor p53 nuclear localization is detected in cells deficient in HSF1 [30, 44]. Moreover, the expression of p53-dependent genes, like *CDKN1A*, was found to be decreased in such cells, thus confirming an influence of HSF1 on the transcriptional activity of p53 as reported by Logan et al. [22]. An effect of HSF1 on p53 nuclear transport may be a consequence of the contribution of HSF1 to microtubule polymerization. It has been reported that HSF1 can stabilize microtubules directly (since it co-sediments with them; [44]) or indirectly by regulating the expression of HSPs and other chaperones that are important for cytoskeleton organization [45]. It has been shown that particularly HSP90 is essential for microtubule polymerization. In HSF1-null mice, a reduced HSP90 expression was found to be associated with a lower expression of the main components of microtubules, i.e., α- and β-tubulins, in epithelial cilia [46]. HSP90 and FKBP4 (HSP56, a member of the immunophilin protein family regulated by HSF1) have also been found to be essential for p53 binding to dynein and its association with microtubules [47].

## Role of HSF1 and p53 signaling in cell fate and aging

Genotoxic and proteotoxic stresses that induce cumulative damage at various levels have been recognized as causal factors for the aging process. When damage is too severe and cells cannot be repaired, they can either trigger cell death programs (through apoptosis or autophagy) or permanently arrest the cell cycle (through a process known as cellular senescence) [48, 49]. Both p53 and HSF1 have been reported to play regulatory roles in stress-induced apoptosis, autophagy and cellular senescence, which in turn affects aging [50, 51]. However, the interplay between both factors in these processes is currently not fully understood and they may even have opposite effects. Activation of p53 or HSF1 may result in enhanced autophagy since both transcription factors are able to regulate core autophagy genes in mammals [52]. In contrast to nuclear p53, however, cytoplasmic p53 may repress autophagy [53]. Wild-type p53 generally promotes apoptosis when DNA damage cannot be repaired [54], while HSF1 is believed to protect cells against apoptosis [11, 55]. Concordantly, HSF1 and HSPs have been found to counteract apoptosis in most cell types, although HSF1 may act as an inducer of apoptosis in heat-sensitive cells (e.g. spermatogenic cells, [56]), which is accompanied by inhibition of HSP synthesis and accumulation of p53 [57, 58].

**Fig. 3:**
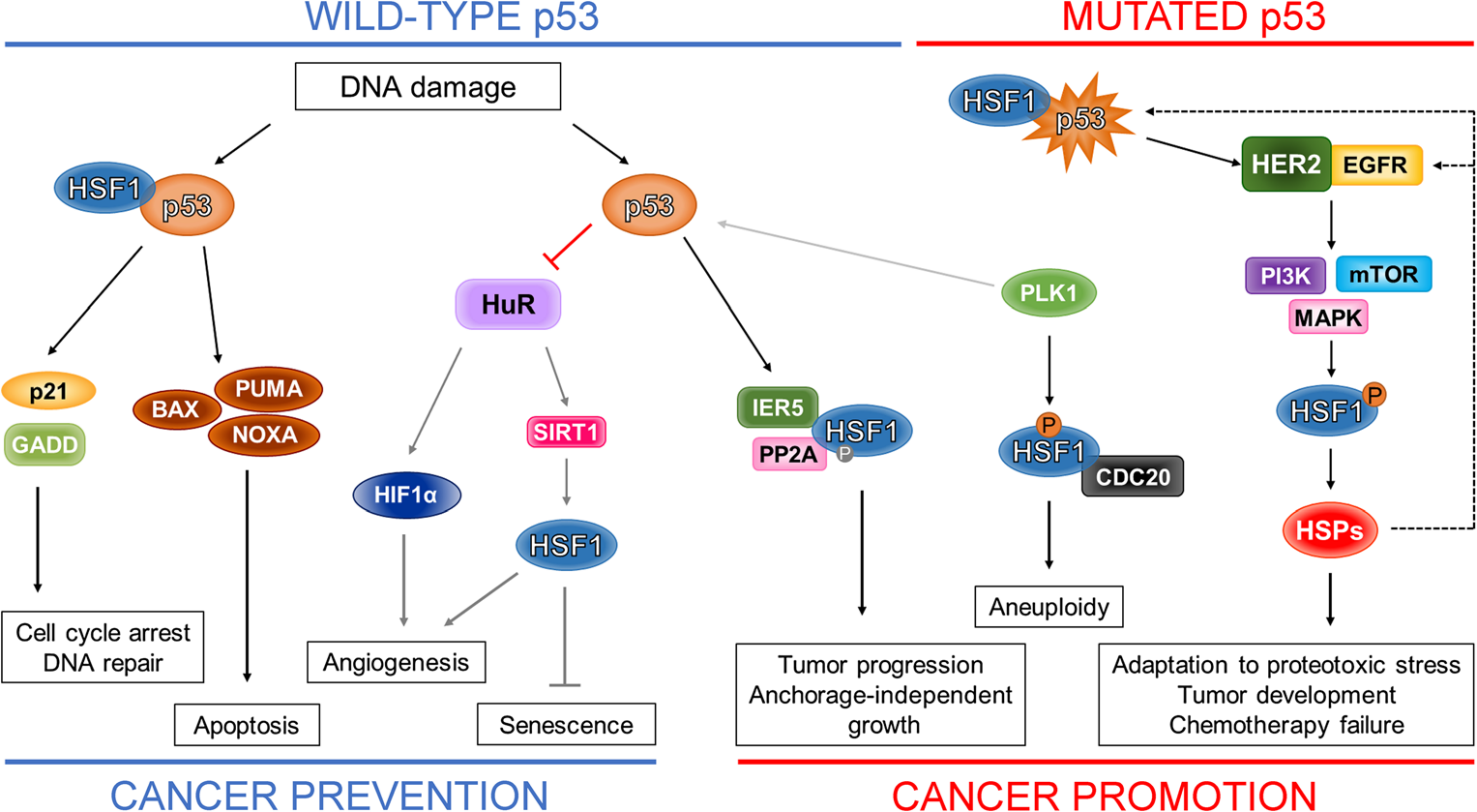
**Crosstalk between HSF1 and p53**. The action of wild-type p53 (e.g. activated by DNA damage) leading to cell cycle arrest and DNA repair, or to apoptosis can be supported/modified by HSF1. Activated p53 can also decrease the expression of ELAVL1 (HuR) which normally stabilizes HIF1α and SIRT1, thereby stimulating angiogenesis and inhibiting senescence. Thus, downregulation of the HuR–HIF1α pathway suppresses angiogenesis while SIRT1 inhibition leads to HSF1 destabilization and senescence induction. On the other hand, wild-type p53 may directly up-regulate IER5 that, in turn, may form a complex with HSF1 and PP2A (protein phosphatase 2A) generating a new HSF1 phosphorylation pattern, which may induce anchorage-independent growth that supports tumor progression. When wild-type p53 is absent in the cell, PLK1 preferentially phosphorylates HSF1 instead of p53, which results in a prolonged interaction of HSF1 with CDC20, cell cycle arrest and aneuploidy (only in cells with mutant p53). In addition, mutant forms of p53 can activate the mTOR, MAPK and PI3K pathways via EGFR/ERBB2, leading to HSF1 activation and induction of HSP expression. Overexpressed HSPs facilitate the adaptation of cancer cells to proteotoxic stress, thereby influencing the development of cancer and the effectiveness of chemotherapy (positive feedback loops are shown by dotted lines)

Similar to its well-known role in apoptosis, wild-type p53 may act as a canonical inducer of cellular senescence [59] (although there are reports showing the opposite; [60]), whereas HSF1 is supposed to relieve cells from it, at least partially [61]. Senescence induced by DNA damaging agents (e.g. during cancer therapy) is associated with activation of the p53–p21 and p38–NF-κB axes of SASP (senescence-associated secretory phenotype) and inhibition of HSF1 activity. Moreover, it has been found that HSF1 depletion alone is sufficient to induce senescence in normal fibroblasts [62]. A positive feedback regulation has been observed. First, activation of the p53–p21 and p38-NF-κB-SASP pathways (induced by DNA damage) has been found to lead to decreased expression of the translation regulator ELAVL1 (ELAV-like protein 1 or HuR, human antigen R), which results in *SIRT1* mRNA decay. Then, reduced SIRT1 (NAD-dependent protein deacetylase sirtuin-1) abundance was found to result in inhibition of HSF1, which further activated the p38–NF-κB–SASP [61] and p53–p21 pathways, partly through dehydrogenase/reductase 2 (DHRS2), a putative MDM2 inhibitor (Fig. 3) [62]. This, in turn, was found to promote senescence. Interestingly, senescence induced after HSF1 depletion was independent of HSPs. Wild-type p53 is also known to inhibit the mTORC1 pathway in a low-energy environment [63, 64], which may restrain the growth of cells experiencing DNA damage. Since mTORC1 is involved in HSF1 phosphorylation and activation [65], it can be speculated that the p53/mTOR pathway may also play a role in the inhibition of HSF1 activity.

## Interplay between HSF1 and p53 in cancer initiation and progression

### Background

Carcinogenesis is a complex multi-step process involving metabolic and behavioral changes enabling transformed cells to survive and adapt to the tumor microenvironment [66]. Most cancers are associated with various genetic and epigenetic changes leading to the malfunctioning of critical genes, including the *TP53* gene. However, cancer cells also depend on the normal functioning of certain genes. As such, activation of specific cytoprotective mechanisms, such as HSR, can support the survival of transformed cells. It has been found that HSF1 normally regulates a subset of genes involved in controlling cell proliferation and cell cycle progression [67, 68]. The requirement of HSF1 also extends to the survival of transformed cells. Consequently, cancer cells may become addicted to HSF1. Although HSF1 is neither a tumor suppressor nor a typical oncogene, it affects many aspects of cellular metabolism that are important for the cancer phenotype, i.e., it modulates signaling pathways associated with growth and proliferation, apoptosis, glucose metabolism, angiogenesis and cell motility [4]. Additionally, its activity has been found to modulate signaling pathways that are altered through the expression of mutant oncogenic proteins, thus affecting the phenotype of cancer cells. This phenomenon has been referred to as “non-oncogenic addiction” [69]. HSF1 has been shown to promote oncogenesis driven for example by mutant p53 (or the loss of 53), mutant RAS, PDGFB (platelet derived growth factor subunit B), ERBB2 (erb-b2 receptor tyrosine kinase 2, also called HER2 or HER2/neu), loss of the tumor suppressor NF1 (neurofibromatosis type 1), and chemical carcinogens [4]. Genome-wide analyses indicate that the activity of HSF1 in cancer cells is strongly associated with metastasis and a poor survival in at least three types of cancer, breast, colon and lung, responsible for ~30% of all cancer-related deaths worldwide [70].

The development of a wide range of cancers (>50%) is associated with mutations in the *TP53* gene. To date, mutations have been identified in over 200 different amino acid positions of the p53 protein. Such mutations impair p53 protein function, which can lead to (i) *“loss-of-function”*, associated with missense mutations that can escalate genomic instability, metastasis, resistance to chemotherapy and radiotherapy, tumor progression and a poor survival, (ii) *dominant negative activity of mutant p53*, which leads to loss of p53 tumor suppressive activity, but the acquisition of dominant negative activity on the remaining wild-type p53 protein, and is associated with accelerated tumor development, (iii) “*gain-of-function*”, which confers oncogenic properties on p53 leading to aggressive tumor growth [20]. Additionally, in those cancers that retain wild-type p53, its activity can be attenuated by several mechanisms, i.e., wild-type p53 can be inactivated by proteins encoded by DNA tumor viruses [71], by down-regulation of p53 cooperators such as ARF, or by overexpression of p53 inhibitors such as MDM2 and its homolog MDM4 (p53 regulator). ARF (also called p14ARF, resulting from an alternative reading frame in the *CDKN2A* locus, which is frequently mutated or deleted in a wide range of tumors) is a tumor suppressor and a key activator of the p53 pathway in response to oncogene activation (e.g. E2F1, MYC, RAS, E1A) [72], whereas MDM2, classified as an oncogene, is amplified in nearly 8% (and MDM4 in ~10–20%, but up to 65% in retinoblastoma) of human cancers with wild-type p53, such as lung, colon, stomach, or breast cancers [73].

### Animal models of p53-dependent oncogenesis and the influence of HSF1

Consistent with clinical data indicating a crucial role of wild-type p53 in the prevention of cancer, mouse models indicate that loss or incorrect p53 function is responsible for the progression of cancer. The first tumors (mostly lymphomas) are observed in p53 null mice at around 10 weeks of age. Although an additional HSF1 deficiency does not prolong the tumor-free survival, it shifts tumor development from lymphomas to testicular carcinomas and soft tissue sarcomas. This phenomenon probably results from inhibition of the production of proinflammatory factors and altered signaling through cytokines. An increase in the frequency of p53-independent apoptosis has also been observed in p53/HSF1 double knockout mice [74]. These results not only suggest a stimulatory effect of HSF1 on neoplastic transformation, but also show a different sensitivity of tissues to HSF1-mediated “non-oncogene addiction“. Studies using mice with the highly oncogenic mutated version of p53 (the structural mutant p53R172H; corresponding to human R175H) have also shown a significant stimulatory effect of HSF1 on p53-dependent carcinogenesis. Heterozygous p53R172H/+ mice (carrying a germline point mutation in one *TP53* allele) with an additional homozygous HSF1 knockout (HSF1−/−) were found to exhibit a longer survival and disease-free time than HSF1+/+ mice. An intermediate effect was observed in heterozygous HSF1+/- mice. The cellular spectrum of transformations was also found to depend on the HSF1 status. HSF1+/+ mice mainly developed sarcomas and lymphomas due to mutant p53 expression, while HSF1+/- mice developed carcinomas, lymphomas and sarcomas in equal frequencies. Importantly, tumors appeared sporadically in mice lacking HSF1 [75]. The mutant p53–HSF1 feed-forward loop was further characterized using an experimental ERBB2-driven breast cancer model [76, 77]. Mutant p53 (R175H) was found to constitutively stimulate ERBB2–EGFR (epidermal growth factor receptor) signaling and to hyperactivate the MAPK and PI3K–AKT–mTOR pathways, which led to transcriptional phosphoactivation of HSF1 on S326. In addition, it was found that mutant p53 directly interacted with activated HSF1 and increased its affinity to chromatin, thereby enabling more efficient HSP expression. The HSPs, in turn, stabilized their oncogenic interactors, including EGFR, ERBB2 and mutant p53. This positive feedback loop allows cells to adapt to an environment of permanent proteotoxic stress and provides cancer cells with superior survival potentials.

It also has to be mentioned that p53 was first identified in a complex with the SV40 large T antigen (TAg) [78, 79]. SV40 TAg can inactivate p53 and RB1, leading to deregulation of the cell cycle and to malignant transformation. Hence SV40 TAg is widely used for immortalizing normal cells [80]. There is convincing data indicating that HSF1 contributes to TAg-mediated oncogenesis. Lack of HSF1 in TAg transformed MEFs (or HEK293T cells) has been found to correlate with increased levels of p53 and RB1, whereas their interactions with the SV40 TAg protein were found to be reduced [33, 81]. Concordantly, HSF1 knockout was found to inhibit the proliferation of transformed MEFs, as well as their tumorigenicity, metastasis and angiogenesis [33].

### Role of p53 and HSF1 in oncogene-induced senescence and angiogenesis

HSF1 has been found to play an important role in evasion of oncogene-induced senescence (OIS), which is critical for the early stages of neoplastic transformation. In experimental breast cancer models, OIS (caused e.g. by PIK3CA, RAS or ERBB2 activation) has been found to be accelerated when HSF1 was silenced by shRNA, resulting in growth inhibition. In this process, OIS is mediated by p21, but only in the presence of wild-type p53 [82]. Otherwise, i.e., in cells expressing both HSF1 and mutant p53, OIS is less effectively induced, which facilitates malignant transformation and tumor growth. Furthermore, it seems that pathways leading to p53-induced senescence may also have inhibitory effects on angiogenesis. Activation of senescence is associated with decreased expression of ELAVL1 (HuR) which, besides inhibition of SIRT1 and HSF1 [61], can affect the translation of HIF1A (hypoxia-inducible factor 1 subunit alpha, HIF1α), a master regulator of angiogenesis (Fig. 3) [83]. Downregulation of the HuR–HIF1α pathway is the main reason why HSF1 knockdown results in suppressed angiogenesis and increased senescence. Importantly, it has also been shown that HSF1 can support the “*gain-of-function*” activities of p53R273H (DNA contact mutant) in the absence of wild-type p53 in breast cancer cells, yet inhibit the clonogenic potential of cells via a wild-type p53-dependent mechanism [84].

### p53 and HSF1-dependent coordination of mitogenic signals by IER5

Another important mediator of the interplay between p53 and HSF1 is IER5 (immediate early response 5) that acts as a regulator of cell proliferation. IER5 expression can be activated through various growth-promoting stimuli [85], but also by genotoxic factors such as ionizing radiation, fluorouracil and doxorubicin, which are known to activate p53 [86, 87]. Induction of IER5 expression by these factors is p53 dependent. In many cancer cells wild-type p53 binds to an active enhancer located 46 kb downstream of the *IER5* gene. Upon DNA damage, due to the formation of a higher-order chromatin structure, p53 localizes in the vicinity of the *IER5* promoter and activates its transcription [87]. Furthermore, IER5 has been reported to act as an activator of HSF1 and to regulate HSF1 activity via a mechanism by which IER5 forms a complex with HSF1 and PP2A (Protein phosphatase 2A) and probably functions as a scaffold allowing PP2A to dephosphorylate HSF1. The hereby generated form of HSF1 (hypo-phosphorylated on S121, S307, S314, T323, S363 and T367) is transcriptionally active, although different from that induced by heat shock (hyper-phosphorylated on S230, S320 and S326) [87, 88]. Thus, genotoxic factors can modify HSF1 activity via wild-type p53 and IER5/PPA2, thereby providing it with a new transcriptional potential. This modified HSF1 may induce a completely different genetic program stimulating tumor progression. In fact, it has been shown by Mendillo et al. [70] that the expression profile of HSF1-dependent genes in breast cells undergoing neoplastic transformation was different from those induced by heat shock. IER5-mediated activation of HSF1 has been found to be required for anchorage-independent growth of cancer cells [87]. Reduced adherence and increased motility of cells with active HSF1 may result from a decreased expression of vinculin, a cytoskeletal protein associated with cell-cell and cell-matrix contacts [89]. Interestingly, a positive feedback loop may exist between HSF1 and IER5, since it is known that heat-activated HSF1 binds to the *IER5* gene promoter and induces its expression [90].

### Impact of HSF1 on genomic instability in cells with mutant p53

One of the basic features of cancer cells, called cancer enabling characteristics, is genomic instability. Aneuploidy, especially structural aneuploidies resulting from chromosome segregation errors, can lead to p53 activation and thereby proliferation arrest [91, 92]. Thus, propagation of structural aneuploidies is limited to p53-deficient cells. Nonetheless, at least a subset of aneuploidies can be propagated also in cells with wild-type p53 [92]. On the other hand, it has been found that a complete absence of p53 did not systematically induce large-scale genomic instability in a rat model [93] as well as in human cells [94]. However, the role of mutant p53 in enhancing genomic instability is well documented, although its underlying mechanisms are not fully resolved yet. Mutant p53 may facilitate the occurrence of structural chromosomal alterations by impairing DNA repair processes as well as the spindle assembly checkpoint [95]. HSF1 has additionally been found to enhance genetic instability in cancer cells carrying a mutant *TP53* gene [96, 97]. In normal cells, wild-type p53 is preferentially phosphorylated by PLK1 (polo-like kinase 1, an early trigger for G2/M transition), whereby the cell cycle is regulated properly [96]. However, in p53 mutant cancer cells with an increased HSF1 expression, PLK1 phosphorylates HSF1 at S216 instead of p53. Excessive HSF1 phosphorylation induces stronger HSF1 interactions with CDC20 (cell-division-cycle protein 20), a protein that normally activates the anaphase-promoting complex (APC), which is required for exit from mitosis. An enhanced interaction of S216 phosphorylated HSF1 with CDC20 results in a reduced affinity of CDC20 to MAD2 (mitotic arrest deficient 2 protein, a component of the spindle assembly checkpoint which prevents aneuploidy arising through improper chromosome separation). This reduced affinity is associated with an increased stability of securin and cyclin B1 and subsequent cell cycle disturbances, which lead to aneuploidy [96, 98, 99].

## p53 and HSF1 as targets for anticancer therapy

The discovery of the dependence of some tumors on one or a few genes, known as “oncogenic addiction”, has led to the development of therapies that target these oncogenes, making the treatment more precise. Such druggable oncogenes are, for example, BCR-ABL in chronic myeloid leukemia, HER2 in HER2-dependent breast cancers, KIT in gastrointestinal tumors, and EGFR and ALK in non-small-cell lung cancer [100]. Since the *TP53* gene is frequently mutated in human tumors [20] it may serve as an attractive target for anticancer therapy. Tumor cells with inactivated or deleted p53 are resistant to many chemotherapeutic agents, of which the action is based on a DNA damage-induced response involving activation of wild-type p53. Hence, strategies to target mutated p53 rely on the restoration of its tumor suppressive functions, the release of tumor suppressive p53 family members (such as TAp73) from complexes with mutant p53, or the induction of mutant p53 degradation (by inhibition of HSPs and reactivation of E3 ubiquitin ligases such as MDM2 and/or CHIP). Such strategies have recently been described in detail by Schulz-Heddergott and Moll [101].

Besides searching for druggable oncogenes, other strategies are also being explored to make cancer treatment more precise and efficient. One such strategy is inhibition of HSPs (described in detail by Chatterjee and Burns [102]). This strategy is based on the observation that cancer cells are more dependent on the chaperoning function of HSPs than normal cells. Overexpression of HSPs in cancer cells is an unfavorable event, as it facilitates survival and adaptation to harsh conditions during toxic anti-cancer treatment. The most commonly used inhibitors are HSP90 inhibitors such as geldanamycin and its analogs. They cause the disruption of complexes of HSP90 with client proteins, such as mutant p53, which in turn inhibits tumor proliferation. Unfortunately, inhibition of HSP90 frequently leads to HSF1 activation and overproduction of other HSPs (i.e., initiation of the HSR), which may promote resistance to cancer therapy [103]. The use of HSP90 inhibitors is often limited by resistance resulting from a P-glycoprotein (ABCB1)-dependent efflux of the inhibiting drugs, and it has been shown that HSF1 can up-regulate the expression of ABCB1 and other ABC transporters [104]. Thus, controlling HSF1 activity may be a better strategy than direct HSP inhibition in cancer cells [105]. To overcome HSP90 inhibitor resistance, combinatory strategies involving HSP90 inhibitors and NSAIDs (Non-Steroidal Anti-Inflammatory Drugs), such as celecoxib and ibuprofen, has been proposed. NSAIDs can sensitize cells to HSP90 inhibitors by inducing both apoptotic and autophagic cell death through inhibition of the AKT/mTOR and STAT3 pathways. This combinatory approach appears very promising since it results in degradation of mutant p53 and down-regulation of HSF1/HSPs and ABCB1 [106].

An alternative strategy to selectively kill p53-mutant cancer cells may be based on targeting a gene that would be synthetically lethal to mutated *TP53*. The phenomenon of synthetic lethality was first detected in *Drosophila* as a lethal combination of perturbations in two genes, where a mutation in either of the genes alone does not affect cell viability. During evolution, many redundancies and feedback loops in cellular signaling have been developed to ensure gene compensation and cell survival when a single gene is perturbed. Thus, inhibition of both compensatory pathways may be an approach to induce cell death due to a synthetic lethal interaction [107]. In the case of p53 loss, cells become dependent on regulation of the G2/M checkpoint that is controlled by ATM, ATR and the checkpoint kinases CHK1 or CHK2. Therefore, inhibition of ATM may sensitize p53-deficient cells to topoisomerase inhibitors and antimetabolites [108], whereas ATR inhibition leads to disruption of the replication checkpoint [109]. Hypothetic genes that are synthetically lethal with an oncogene or a tumor suppressor gene are not necessarily affected in cancer cells. Moreover, cells that have lost a tumor suppressor gene may have become addicted to functions of the non-oncogenic gene [69].

Cancer cells may be addicted to HSF1 that is overexpressed due to proteotoxic stress in the tumor microenvironment. Hence, HSF1 may serve as a promising target for strategies based on the concept of synthetic lethality. Activation of HSF1 can also be triggered by genetic changes (such as loss of the tumor suppressor NF1 [110]), by altered kinase signaling [4], or by p53 “*gain-of-function*” mutations [111]. Ablation of HSF1 has been shown to selectively suppress lymphomas arising in p53−/− mice. Moreover, an increased frequency of p53-independent apoptosis has been observed in p53/HSF1 double knockout mice [74]. HSF1 targeting may also serve as a potential intervention strategy to prevent the development of drug resistance. Promising experimental results have been obtained for ERBB2 (HER2)-positive tumor treatment (in which mutant p53 cooperates with HSF1 generating a positive feedback loop). Targeting of HER2 in breast cancer cells (e.g. by lapatinib) has been found to lead to a decrease in HSP90 expression by reducing the HSF1 transcriptional activity and, consequently, to MDM2 and mutant p53 protein degradation [112]. However, the use of lapatinib is often associated with resistance due to the presence of compensatory receptor tyrosine kinase (RTK) pathways. Intriguingly, lapatinib-resistant cells are characterized by activated HSF1 and HSP overexpression. In this case, pharmacological HSP90 or HSF1 inhibition was found to lead to a downregulation of HER2 and mutant p53, which resulted in a decrease in lapatinib resistance [113]. Therefore, considering the existing crosstalk between p53 and HSF1, targeting of HSF1 appears to be a promising strategy for the treatment of p53-mutated cancers.

## Concluding remarks and perspectives

The molecular complexity of HSF1- and p53 (wild-type or mutant)-dependent signaling pathways makes their interactions multifaceted and dependent on specific cell type contexts and signal activation. The most studied relationship between HSF1/HSPs and p53 concerns the dependence of the stability and activity of p53 on the HSP chaperone function. HSF1 generally supports the proper action of wild-type p53 in transcriptional regulation of the cell cycle. However, HSF1 and p53 may have opposite effects on apoptosis or cellular senescence. Importantly, senescence mediated by wild-type p53 results in HSF1 downregulation, which is associated with growth retardation and angiogenesis inhibition, thus preventing tumorigenesis. *TP53* is mutated in approximately half of all tumor cases, while somatic mutations in *HSF1* have so far not been recorded in human tumors. Although increased HSF1 expression is not causative for tumorigenesis, HSF1 is frequently overexpressed in cancer. Importantly, HSF1 not only supports the action of wild-type p53, but also its oncogenic mutant forms and generally provides a superior survival of cancer cells. Senescence induction is decreased while angiogenesis is increased in cells with mutant p53 and HSF1 overexpression. Additionally, HSF1-mediated cell cycle disorders leading to aneuploidy have been observed only in cells with mutant p53. The interplay between p53 and HSF1 affects the response of cancer cells to anticancer treatment. In particular, “*gain-of-function*” p53 mutants cooperate with the heat shock response machinery, which introduces an adaptive mechanism to enhance tumor cell survival, making them more resistant to proteotoxic stress. Therefore, inhibition of HSF1 (in parallel to other treatments) may be a promising strategy for overcoming drug resistance in cancer cells, thereby enhancing the efficacy of treatment and reducing undesired side effects.
